# Swarming Behavior in *Anopheles gambiae* (sensu lato): Current Knowledge and Future Outlook

**DOI:** 10.1093/jme/tjab157

**Published:** 2021-10-07

**Authors:** Rowida Baeshen

**Affiliations:** Faculty of Sciences, Biology Department, University of Tabuk, Tabuk, Kingdom of Saudi Arabia

**Keywords:** malaria vector, assortative mating, mating behavior, swarming ecology, wing beat

## Abstract

Effective management of insect disease vectors requires a detailed understanding of their ecology and behavior. In *Anopheles gambiae* sensu lato (s.l.) (Diptera: Culicidae) mating occurs during swarming, but knowledge of their mating behavior under natural conditions is limited. Mosquitoes mate in flight over specific landmarks, known as swarm markers, at particular locations. Swarms consist of males; the females usually approach the swarm and depart following copulation. The number of mating pairs per swarm is closely associated with swarm size. The shape and height of swarm markers vary and may depend on the environmental conditions at the swarm’s location. Male–male interactions in mosquito swarms with similar levels of attractive flight activity can offer a mating advantage to some individuals. Flight tone is used by mosquitoes to recognize the other sex and choose a desirable mate. Clarifying these and other aspects of mosquito reproductive behavior can facilitate the development of population control measures that target swarming sites. This review describes what is currently known about swarming behavior in *Anopheles gambiae* s.l., including swarm characteristics; mating within and outside of swarms, insemination in females, and factors affecting and stimulating swarming.

*Plasmodium* species., the causative agents of malaria, are transmitted through the infectious bite of a female mosquito. *Plasmodium falciparum*, *P. vivax*, *P. ovale*, *P. malariae*, and *P. knowlesi* are the five *Plasmodium* species known to cause malaria in humans (Cox-Singh and Singh 2008). Worldwide, approximately 229 million cases of malaria were reported in 87 endemic countries in 2019 and there were 409,000 deaths from *Plasmodium* species infection (Table 1) (World Health Organization [WHO] 2020). About 94% of these deaths occur in Africa (WHO 2020) where there are more than128 species of *Anopheles* (Kyalo et al. 2017), with *Anopheles coluzzii*, *An. gambiae* (sensu stricto), and *An. funestus* being the most common vectors (Takken and Lindsay 2019). The estimated number of malaria cases and deaths caused by *Plasmodium* species infection in five regions in 2019 is shown in Table 1 (WHO 2020).

**Table 1.** Estimated number of malaria cases and deaths in five regions caused by infection with *Plasmodium* species in five world regions in 2019^*a*^

Number of malaria cases (×10^3^)	Number of malaria deaths	Region
215,000	384,000	Africa
6300	9000	Southeast Asia
5200	10,100	Eastern Mediterranean
1739	3200	Western Pacific
889	551	America

^*a*^ WHO (2020) data.

**Fig. 1.**
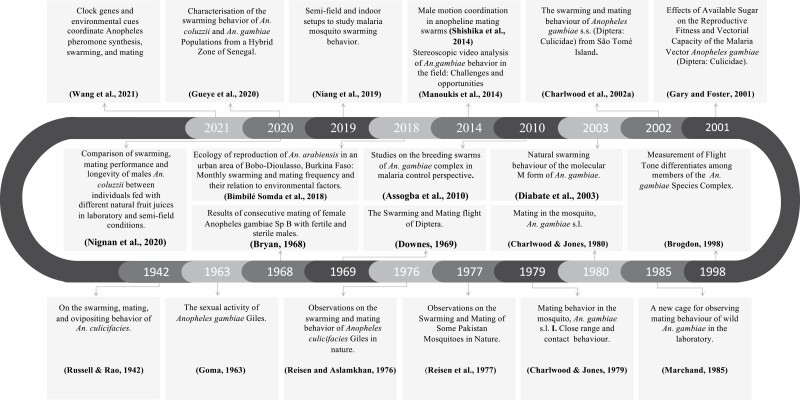
The major discoveries regarding swarming in Anopheles from 1942 to the present are summarized by topic.

Between 2002 and 2017, vector techniques were the primary tool used to control malaria spread. Long-lasting insecticidal net programs and indoor residual spraying were critical in reducing the spread of the disease (Knols et al. 2016, Barreaux et al. 2017). However, there are two main problems with these methods: first, whether used alone or in combination, they do not effectively reduce malaria incidence in high-transmission areas; second, insecticide resistance is widespread and increasing among the major malaria vectors in Africa (Knols et al. 2016, Benelli and Beier 2017). Another major issue is that these strategies mostly affect *Anopheles* species that shelter indoors (e.g., in homes) and feed at night. Changes in mosquito host choice and feeding time, as well as a shift to outdoor feeding due to the modification of behavioral responses, have necessitated the development of more effective and environmentally sustainable vector control strategies to complement existing ones (Nyasembe et al. 2014, Barreaux et al. 2017). Diabaté and Tripet (2015) identified two possible control methods that focus on male swarming behavior: creating a trap based on sounds, chemicals, or other sensory cues used by *An. gambiae* in swarm formation, and using a lure-and-kill strategy that exploits visual cues associated with swarming. Additional control strategies depend on understanding and manipulating mating behavior such as the sterile insect technique (SIT), which is currently being studied for *An. arabiensis* (Helinski and Knols 2008, Alphey et al. 2010). SIT involves the mass release of sterilized males into wild populations; females that mate with these males produce infertile eggs, leading to a reduction in population size. Although the SIT has been used in various insect pests, it has not been particularly effective against *Anopheles* (Benedict and Robinson 2003).

Mating in *An. gambiae* sensu lato (s.l.) occurs primarily in swarms. However, this behavior is poorly understood due to the difficulty of observing the rapid movement of flying insects. Moreover, mating takes place at dusk, which makes it difficult to track where and when it has occurred (Charlwood and Jones 1980). Consequently, only a small number of studies on the mating behavior of *An. gambiae* s.l. under natural conditions have been published, and it is not well understood.

Successful swarming can result in high mating rates, reflecting the high reproductive investment of *An. gambiae* s.l. Clarifying the reproductive biology of *Anopheles* can identify the specific reproductive goals that can be targeted by mosquito population control measures (Shaw et al. 2015). In one study, targeting the males of *An. gambiae* s.l. in swarms with a mixed carbamate and pyrethroid aerosol decreased their reproductive rate by killing many males and visiting females, leading to an 80% reduction in population size (Sawadogo et al. 2017). The authors also observed that the female insemination rate greatly declined, and there was a marked shift in the age profile of the males to younger individuals that were incapable of mating. This represents a major shift from existing and earlier malaria vector control methods, which have mainly involved killing female mosquitoes (Sawadogo et al. 2017). The inability of these control measures to completely halt transmission can be attributed to various factors, including insecticide resistance, taxonomic and behavioral variability across mosquito species, and populations that exhibit nonnormal or atypical resting and feeding behaviors (Sawadogo et al. 2017).

New technologies, such as stereoscopic image analyses, used to locate and track swarming mosquitoes in the field, are extremely useful for meeting the abovementioned objectives, as they allow researchers to directly observe, record, and quantify mating behavior (Butail et al. 2012). A better understanding of the relationship between swarming habits and reproductive behavior in mosquitoes can guide the development of successful management programs (Cabrera and Jaffe 2007) that would alleviate the burden and threat of mosquito-borne diseases.

This review summarizes what is currently known about swarming behavior in *An. gambiae*, including its swarm characteristics, mating within and outside of swarms, insemination of females, and factors affecting and stimulating swarming.

## Mosquito Swarming Behavior

The word ‘swarm’ has been used to describe a variety of insect aggregations (Clements 1999). *Diptera* species engage in swarming behavior as part of their mating process (Downes 1969, Yuval 2006), and the same is true for various species of *Anopheles*, including *An. gambiae* s.l. (Charlwood et al. 2002b, Yuval 2006, Manoukis et al. 2009, Shaw et al. 2015, Achinko et al. 2016).

Swarming consists of several characteristics (Clements 1999). First, insects in swarms fly in loops or zigzags within a limited space in locations with specific environmental features. Second, swarms typically consist of males; when females enter a swarm, the males track and try to mate with them. Third, swarming occurs at a specific time of day in each species, generally around dawn or sunset. It is widely believed that swarms facilitate mating in mosquitoes (Downes 1969, Reisen and Aslamkhan 1976, Baker et al. 1980, Sullivan 1981, Bock et al. 1983, Clements 1999, Yuval et al. 1993) and are an essential step in the mating process (Yuval 2006, Howell and Knols 2009, Shaw et al. 2015).

## Swarm Characteristics

### Swarm Number and Shape

Numerous culicid dipterans mate in swarms. The number of individuals in a swarm range from 10 or less to thousands of flying males (Clements 1999). The number of males increases within a few minutes of swarm formation (Russell and Rao 1942). For example, the average number of *An. maculipennis* var. *atroparvus* males (Diptera: Culicidae) in a swarm is usually between 25 and 50 but can sometimes increase to more than 1,000 (Cambournac and Hill 1940). In contrast, the number of swarming males is less than 500 in *An. gambiae* sensu stricto (s.s.) and ranges from 100 to 1,500 in *An. melas* (Assogba et al. 2010). The estimated number of males per swarm and number of females entering swarms can also vary markedly (Dao et al. 2008). However, the number of individuals in any given swarm tends to remain the same over time (Charlwood et al. 2003). The factors contributing to differences in the number of individuals of both sexes in a swarm are unknown, but it may include environmental variables, such as wind, sunlight, and the presence of predators or other organisms that disturb the swarm, as well as local geographic features.

In the early morning or evening, solitary males fly in a zigzag pattern at certain locations (Russell and Rao 1942; Reisen et al. 1977, 1985); however, *An. gambiae* males also aggregate into a spherical swarm (Marchand 1984). *An. gambiae* males adjust their position inside the swarm to improve their probability of mating with an incoming female (Diabaté et al. 2011). Female mosquitos might detect the size of a swarm visually (Diabaté et al. 2011). For male *An. gambiae*, swarm size likely has a positive influence on mating outcome (Diabaté et al. 2011). The number of mating pairs and total number of individuals in a swarm, swarming period, and first mating vary across season and according to the monthly rainfall. Predators (e.g., bats and dragonflies) can disrupt mating, thus decreasing the number of mating pairs (Sawadogo et al. 2014). The number of mating pairs per swarm is positively correlated with swarm size (Bimbilé Somda et al. 2018). More research is needed to determine the mating success of individual males concerning their quality and swarm size. In addition, more studies are needed to clarify the relationship between swarm size and environmental factors such as wind, temperature, and gravity.

### Swarming Period (Start, End, and Duration)

Male mosquitos mate once after sexual maturity and only when their terminalia is inverted and their antennal fibrillae are completely erect (Charlwood and Jones 1979). Inversion of the terminalia occurs 24 hr postemergence (Clements 1999), and the proportion of male *An. gambiae* capable of mating increases 1–3 d after emergence (Charlwood and Jones 1979). *An. arabiensis* males are able to effectively mate just 11 hr after emergence, and 42% of males have already completed genitalia rotation at this point. At 17 hr postemergence, the genitalia of 96% of laboratory-reared males have completed development (Oliva et al. 2011).

Swarms of *An. gambiae* and *An. arabiensis* begin about 10 min after sunset and lasts for approximately 20 min. (Marchand 1984). Similarly, *An. melas* and *An. gambiae* s.s. adults spend about 10–20 min swarming in the field (Assogba et al. 2010). In *An. funestus*, the mean swarming period is 12.9 min (Kaindoa et al. 2019). Females spend approximately 30 s in swarms before mating with a male, although the duration increases under strong moonlight (Charlwood and Jones 1980). Swarm timing rather than duration may be critical for mating success in male Anopheles, with the highest number of matings associated with high swarming activity (Charlwood et al. 2002b, Diabaté et al. 2003).

Swarming time varies significantly over the year. For instance, in late July to early October in Burkina Faso (West Africa), swarming begins after sunset, but it occurs before sunset from late October to early July (Bimbilé Somda et al. 2018). Light levels at the start of swarming are inversely related to temperature, but not to relative humidity (Reisen et al. 1977). Swarms can form recurrently over the same site in a single season or across many seasons (Diabaté et al. 2009, 2011; Sawadogo et al. 2014, 2017; Kaindoa et al. 2017). It is unclear why males form swarms that enter by females that reach the area later, as males create swarms for mating, so it is not surprising that they would form swarms for any time females are around. The females may communicate their location to potential mates via visual (markers), olfactory (chemical signal), or auditory (wing beats) cues that increase the probability of mating.

### Swarm Location

One of the stimuli used to establish swarm location is a swarm marker. These are typically dark–light contrast points on the ground or horizon that are used for orientation (Howell and Knols 2009). Markers at a swarming location may be visual (Yuval 2006), although not all types of swarming insects use visual cues (Clements 1999). Some combination of light/dark and ground-based characteristics that attract both sexes can be markers. It is unknown why males are attracted to these landmarks, although visual cues play a crucial part in swarming site selection (Diabaté and Tripet 2015).

Identifying swarm markers is challenging, or even being sure that they exist because many natural ones are not visible to the observer (Howell and Knols 2009). In addition, it remains unclear whether mosquitoes use a single marker or a combination of several markers. Several types of markers can correspond to different locations (Howell and Knols 2009). *An. gambiae* s.s. swarm mainly above bare ground, whereas *An. coluzzii* swarms over physical objects including wood, waste, and grass that form a dark–light contrast against the ground (Gueye et al. 2020). It has been suggested that such markers can be used to differentiate between *An. coluzzii* and *An. gambiae* s.s. swarms (Gueye et al. 2020). Visual markers on the ground not only shape swarms but also serve as landmarks to establish and maintain the position of the swarm when the marker is moved or, as more occurs often in the field, when the swarm is disrupted by wind or predators (Poda et al. 2019). In both *An. coluzzii* and *An. gambiae* s.s., large markers attract more mosquitoes but have different effects on swarm characteristics; *An. coluzzii* swarm size increases both vertically and horizontally, while *An. gambiae* swarm size increases only horizontally and are further above the ground (Poda et al. 2019).

The markers most frequently used by *An. coluzzii* are wood stacks, bare earth, sheds, wells, and manure stacks (Sawadogo et al. 2014); these are characterized by contrasting dark/light patterns, as in the case of the intersection of a plant (dark) and footpath (light) (Diabaté et al. 2009). By contrast, swarms of *An. gambiae* s.s. have only been observed above galleries, manure, and waste piles (Sawadogo et al. 2014), or bare land (Diabaté et al. 2009). These are the most commonly reported markers; however, they are not limited to either type throughout Africa.

*An. arabiensis* swarms are classified according to their height between 1.5 and 4.5 m around breeding sites and above the roofs of houses (Dabire et al. 2014). Most *An. funestus* swarms occur over bare ground or lawns near houses at a mean height of 1.7 m (Kaindoa et al. 2019), or over sandy clearings near houses on successive evenings at a height of 2–4 m (Gueye et al. 2020). The average height of swarms is 1.80 m for *An. gambiae* s.s. and 1.62 m for *An. coluzzii* (Gueye et al. 2020). Swarm height is influenced by visual markers, with the highest swarms occurring above houses or roofs of buildings and the lowest above open land (Dabire et al. 2014).

*An. gambiae* s.s. and *An. coluzzii* swarms have most if not all of the characteristics of leks (Alcock 1987). Swarming takes place over locations that have no resources that females can exploit and are only used for mating. Females can choose between males in the swarm, and intense male–male interactions occur within the swarm in the competition for females. Females have the opportunity to evaluate several males before mating (Butail et al. 2012, Shishika et al. 2014).

The specific position within a swarm that an individual male occupies can improve its chances of mating if it is the one most visited by females or the one that provides the best access to arriving females. The swarms’ centers are usually about 100 cm above the ground (40–200 cm) (Marchand 1984). The highest density of individual *An. gambiae* are found near this center point. This high density may result in individual males optimizing their chances of encountering a female; it provides them with the quickest access to any part of the swarm’s perimeter if a female enters the aggregation (Manoukis et al. 2009). Alternatively, it could result from mosquito orientation via cues within the swarm. For example, females may fly toward a swarm based on sound; this cue may induce them to pass through the center more frequently. In addition, females may be more likely to move through the middle of the swarm, which makes this location favorable to waiting males. Finally, Manoukis et al. (2009) reported that swarming males are aware of both other males and swarm markers, but it is uncertain where females find males to mate within the swarm. Few studies have investigated the geographic distribution of saltwater mosquitoes (*An. melas*) in West Africa (Charlwood and Jones 1980, Coetzee et al. 2000), and their swarms have not been characterized. Swarms of *An. melas* have mainly been observed over barren land near or within salt-producing sites. Swarms of *An. melas* were not present throughout the rainy season. A significant pattern of spatial segregation has been identified between *An. coluzzii* and *An. melas* swarms, indicating that the two species share unique species-specific mating units (Assogba et al. 2014).

## Mating Within and Outside of Swarms

Most data on the mating behavior of *An. gambiae* are derived from field studies, as it is difficult to recreate swarms of this species in large field cages or in the laboratory (Knols et al. 2002). Although laboratory observations of swarming mosquitoes under controlled conditions can provide valuable information, small laboratory cages (30 cm^3^) are not suitable for eliciting swarming flight (Facchinelli et al. 2015). As such, few studies have considered *An. gambiae* s.s. swarming in the laboratory. In one study, an artificial horizon with a bright mock sky was used to stimulate the swarming of *An. gambiae* s.s. and *An. arabiensis* in cages less than 1 m^3^ (Marchand 1985); in an earlier study, male swarming was induced in a 1.7-m^3^ cage (Charlwood and Jones 1980). Such so-called mesocosm cages have been improved but since then have only been used to evaluate the effects of sugar on mating performance in *An. gambiae* rather than swarming behavior (Stone et al. 2009, Jackson et al. 2015).

Swarming was recently examined at the Mosquito Ecology Research Facility (MERF) in a semifield enclosure with 12 sections (L × W × H: 10.0 × 6.0 × 4.5 m) exposed to constant environmental conditions (Niang et al. 2019). The results suggested that using this type of system can provide useful data on mosquito ecology and mating behavior. Swarming has also been observed in adult populations in a semifield setting (SFS) consisting of large field cages (21 × 9.1 × 7.1 m) in a natural environment. Because SFS mosquito populations are established directly from the field or in the laboratory, the vector’s host preference is not known. Thus, while the SFS can bridge laboratory and fieldwork, it cannot replace field studies (Ng’habi et al. 2010). Nonetheless, this technology can benefit researchers and vector control specialists who seek to develop and implement techniques to control mosquito populations. The relevance of laboratory and semifield data to the real world is an important issue to address and requires quantitative analyses.

Under field conditions, *An. gambiae* females copulate once in their lifetime (Bryan 1968, Goma 1963). However, in the laboratory environment, females are inseminated more than once, as evidenced by the deposition of several mating plugs and their active attempts to reject males after a previous mating event (these observations were mostly performed on females 2 d after the first mating; Charlwood and Jones 1979). The presence of external mating plugs indicates that an inseminated female in flight is unable to prevent subsequent males from attempting to copulate with her (Charlwood and Jones 1979). It is unclear whether females in the field return on successive nights to the same mating swarm. The rapidity of mating pair formation implies that neither the male nor the female engages in courtship or selection. On the other hand, considering that females mate only once and their overall reproductive fitness depends on a single partner, a lack of selection on the part of the female appears unlikely (South and Catteruccia 2016). Male reproductive performance within the swarm can be enhanced by identifying and aligning with females more quickly than competitors, although the role of postcopulatory competition in male reproductive success is unclear (Cator et al. 2021).

Over time, mating experiments using laboratory strains have modified the mating phenotype of the male to the point of altering its insemination ability and the size and shape of the mating plug passed to females, with potential effects on sperm uptake and survival. Inbreeding has greatly affected older strains (KIL and Mopti 2003 strains, which have been established for 35 and 8 yr, respectively), resulting in male sterility and a dramatic decline in male and female fecundity (Ekechukwu et al. 2015). It would be useful to analyze the mating behavior of released, genetically modified males and how this affects population control strategies such as the SIT.

## Insemination of Females

Females are inseminated in two steps: the male gonopore pushes against the female spermathecal duct, and the male aedeagus is inserted into the female vagina to transfer sperm and accessory gland secretions (Spielman et al. 1974). Five or more females can be inseminated by a single *An. gambiae* male (Giglioli and Mason 1966, reviewed by Clements 1999).

If a mating plug is found in the atrium, the spermatheca usually has sperm. Therefore, females who do not receive a mating plug cannot store sperm, which has profound implications for fertility. The mating plug is thus essential for sperm preservation and effective insemination (Rogers et al. 2009). However, the plug offers limited protection against subsequent male sperm storage (Rogers et al. 2009). Seminal fluid proteins send chemical signals to the female nervous system, causing her behavior to change and reproductive hormones to be released (Chapman 2009). During sexual inactivity, the seminal vesicles and accessory glands are replenished with sperm and secretions, respectively (Mahmood and Reisen 1982).

Male accessory glands in many mosquito species take several days to mature, which is required for effective sperm transfer (Clements 1999). Hence, optimal mating in *An. gambiae* s.s. and *An. arabiensis* occurs in 5- to 7-d-old males (Verhoek and Takken 1994). There is limited information on female insemination rates and the effects of swarming activity. Females often exhibit swarming-like behavior that is contingent on their insemination state, with inseminated females being less likely to swarm in both laboratory and semifield environments (Poda et al. 2019). Mating behaviors in *An. gambiae* are not well understood (Charlwood et al. 2002a, Diabaté et al. 2011, Dabiré et al. 2013, Sawadogo et al. 2014), and their molecular basis requires further investigation (Thailayil et al. 2011, Shaw 2014).

Laboratory-reared mosquitoes produce smaller sperm and have larger testes and smaller accessory glands than field-collected males; in fact, sperm length decreases with laboratory colonization time (Baeshen et al. 2014). An increase in testis size is associated with smaller accessory glands, suggesting that the size of this reproductive organ quickly decreases through selection in the laboratory environment (Baeshen et al. 2014). Thus, there are major differences in the morphology of laboratory and natural mosquito populations, highlighting the need for more comparative studies on the mating ecology of mosquitoes under artificial, semifield, and field conditions (Baeshen et al. 2014). Mate selection during swarming may depend on several factors including fast flight, sound, body size, or a combination of several factors, including assortative mating (Jaffe 2002).

Sperm acquisition by a female mosquito is a possible target for vector control strategies. In *An. gambiae* females, mating permanently disables their receptivity to further insemination by other males and stimulates oviposition (Clements 1999, Tripet et al. 2003). Given this dependence on a single mating event for lifetime reproductive success, interfering with insemination or oviposition can significantly affect the size of natural mosquito populations. Fertility is targeted by natural insect pest control techniques such as the SIT (Knipling 1955). A better understanding of mating and other aspects of *Anopheles* fertility can improve the performance of the SIT and reveal new biological targets for interventions (Baldini et al. 2012). Unfortunately, while traditional SIT is more acceptable to the public than other transgenic methods, the high fecundity of mosquitoes has undermined long-term suppression programs (Benedict 2021).

## Factors Affecting and Stimulating Swarming in *Anopheles*

### Roles of Antennae and Wing Beats in Swarming and Mating Behavior

The flight tone produced by mosquito wing beats is subject to sexual selection, and several harmonics have been identified in Johnston’s organ of the antenna pedicel (Clements 1999). Flight tone is used by mosquitoes to recognize the other sex (Hartberg 1971, Clements 1999). Differences in flight tone could serve as an isolating mechanism for reproduction (Cator et al. 2010) or may have another behavioral function (Brogdon 1998).

Male mosquitoes erect their antenna hair when they begin to swarm to detect female wing beats (Nijhout and Sheffield 1979). Females and males alter their wing beat frequencies so that they match one another, leading to harmonic convergence between members of the same species (Gibson et al. 2010, Pennetier et al. 2010). The time taken for a swarm to reach harmonic convergence varies with the body size of potential partners (Cator et al. 2010). Thus, the contribution of flight tone to the process of sexual selection should be assessed in terms of wing beat frequencies in free-flying swarms (South and Catteruccia 2016).

In mating swarms of *An. coluzzii* and *An. gambiae* s.s., male–male interactions mostly involve collision avoidance, but parallel flight between mating pairs within a swarm is a frequent occurrence and may reflect each male matching his velocity to that of a female (Shishika et al. 2014). It is unclear whether flight tone frequencies differ between laboratory and field populations; any differences will be important, as future studies will likely rely on laboratory data to evaluate the effectiveness of pest control measures involving the release of transgenic or sterile males into the wild (Knols et al. 2007).

Male mosquitoes respond to female flight tones over distances between 5 and 30 m. and leave swarms to pair with nearby females, flying in and out of the swarm with the females that join (Charlwood and Jones 1979). Behavioral and physiological investigations have shown that *Aedes aegypti* can hear and utilize low-frequency tones from a distance upto ten m. In addition, *Ae. aegypti* is sensitive to sound frequencies ranging from 150 to 500 Hz (Menda et al. 2019). The frequency ratio of the swarming sound made by *An. coluzzii* and *An. gambiae* s.s. males may be loud enough to be heard by *An. coluzzii* females at least 3 m away from the swarm’ center. Females have a hearing threshold that is closer to 48 dB (sound pressure level (SPL) than 36 dB SPL. As a result, acoustic communication between mosquitos is limited to dyad encounters at close range (Feugère et al. 2021). Females may use the nearby sound of a chasing male to prevent being inseminated by the wrong species. However, further study should focus on long-range cues such as vision or olfaction (Feugère et al. 2021).

### Assortative Mating and Swarming

Swarms play an essential role in the mating system of *An. gambiae* by providing a mating arena for conspecific females and males to select possible mates, i.e., intraspecific sexual selection. On the other hand, they serve a crucial role in premating reproductive isolation between sibling species and forms. Therefore, females and males can correctly choose conspecific partners when swarming, selecting potential mates, and exiting the swarm in copula (Diabaté and Tripet 2015). Knowing the processes that occur in these steps is critical because it could lead to alternative approaches of enticing, trapping, and killing females or males (Diabaté and Tripet 2015).

Premating separation is a type of sexual isolation, in which individuals of different species are less attracted to one other; it can include ecological differentiation or any other attribute that makes them less likely to mate (Ritchie and Immonen 2010). The most typical driving forces of sexual isolation in closely related insects are differences in sexual behavior such as courtship or complex phenotypes and associated preferences (Ritchie and Immonen 2010).

*An. gambiae* uses audio–motor interactions to detect different tones, which occur reliably between a male and a virgin female of the same form (M form [*An. Coluzzii*] and S molecular form [*An. gambiae* s.s.]). The different tones created by nonlinear oscillations of the antennae of a pair of mosquitoes and recognized by the Johnston’s organ are the key to frequency matching. Mosquitoes can match flight-tone harmonic frequencies over their aural range by adjusting their wing beat frequency. *An. gambiae* matches flight tones at a frequency outside the range of Johnston’s organ syllabic reactions to auditory stimulation (Pennetier et al. 2010).

The use of hearing by males to locate females when they enter swarms is evident; nevertheless, the mechanisms that influence females’ acceptance or rejection of copulation are poorly understood (Tripet et al. 2004). Wing beat increases with temperature, age, and size, as does flagellum sensitivity. Strong premating isolation cannot be explained by hearing alone (Tripet et al. 2004). It is uncertain whether harmonic convergence occurs because a male and female who are initially attracted to each other attempt to meet in-flight and copulate. If this is true, it is unclear what signal(s) account for the initial attraction. They could be qualitative flight tone that are changes indicative of vigor, size, or they could be other signals (Diabaté and Tripet 2015).

Diabaté et al. (2009) highlighted the importance of ground markers as a predictor of swarm segregation among molecular forms of *An. gambiae*. Because spatial swarm segregation is nearly complete in forms found in Mali and Burkina Faso (West Africa), it most likely contributes significantly to assortative mating between the forms. However, this does not rule out the possibility that more than one mechanism of recognition occurs across the range of molecular forms. Gueye et al. (2020) illustrated the role of swarm markers in determining swarm segregation between *An. coluzzii* and *An. gambiae*.

Mating investigations can help elucidate reproductive isolation in connection with genetic polymorphism in various species (Coluzzi et al. 1979). Thus, studying mating behavior in the malaria mosquito may provide a means of understanding mechanisms of reproductive isolation between *An. gambiae* molecular forms (Lanzaro and Tripet 2003) and between the seven sister species to *An. gambiae* s.l. (Marchand 1984).

Molecular and genetic research has revealed that the hybridization rate between *An. coluzzii* and *An. gambiae* s.s. is not significant in most of their sympatric distribution range (della Torre et al. 2005, Gueye et al. 2020), demonstrating positive assortative mating over their wider ranges (Coetzee et al. 2013). The ecological conditions that result in rare *An. gambiae* s.s. in populations dominated by *An. coluzzii* may encourage the breakdown of spatial swarm segregation and assortative mating between the two species. The low average hybridization rates found in the larvae and adult indoor stages relative to cross-mating rates support the notion that postmating selection processes operating on hybrids may happen mostly before and/or during the young larval instars (Niang et al. 2015).

Furthermore, spatial swarm segregation is one of the best-described mechanisms of premating reproductive isolation (Diabaté et al. 2009). No connection has been discovered between swarming behavior and hybridization (Gueye et al. 2020). However, it may be that mate recognition in a swarm is more significant than swarm segregation because the number of mixed swarms appears to be too great to explain the low frequency of cross-mating and hybrids (Diabaté et al. 2006). The mechanism through which the sexes are attracted to each other may lead to specific mate recognition systems that help avoid hybridization. The mechanisms underlying assortative mating, when males and females prefer to mate with partners who have similar features, in *An. gambiae* remain unknown

Studies of *Anopheles* have reported assortative preferences for body size (Diabaté and Tripet 2015). However, in such studies, only a single size class of male or female has been explored (Diabaté and Tripet 2015). In *An. gambiae*, male body size plays a critical role in swarming and mating. In one study, varied body sizes were created in males by feeding larvae three amounts of food (10 mg, 20 mg, and 40 mg) (Ng’habi et al. 2008). Although males of intermediate size had better success mating during swarming than larger or smaller males, their average survival was 15% lower than that of the other two groups. Thus, while evidence suggests that larval nutrition and subsequent body size play an active role in mating success, the relationship between this and survival in different phenotypes is complex (Ng’habi et al. 2008). Cator et al. (2010) investigated harmonic convergence behavior in *An. gambiae* and discovered that flight tone frequency varies with size, such that larger individuals have significantly higher flight tones. Both males and females have a shorter latency to higher frequency tones, indicating that both sexes use sound to determine the size and thus fecundity in potential mates (Cator et al. 2010). The extent of assortative mating in nature and its proportional role in determining male mating success are unknown (Cator et al. 2021).

Studies of indoor mating cast light on critical elements of *An. gambiae* mating behavior. In *An. gambiae* and *An. coluzzii*, swarming may occur inside (11%) houses, but is typically outside (89%) (Gueye et al. 2020). Indoor environments are more suitable for *An. arabiensis* and *An. coluzzii* than for *An. gambiae* s.s.; approximately 90% of females remain virgins under this condition, suggesting that they prefer mating in outdoor swarms. Indoor mating may have developed to offset the limited ability of certain males to mate in swarms (Dao et al. 2008). Further studies on indoor mating strategies under different conditions are needed to determine their relative contribution to variation in population density, including across seasons (Tripet et al. 2004). In addition, comparing mosquito behavior in indoor vs outdoor settings can clarify biases associated with laboratory-based research (Clements 1999). Other as-yet unexplained elements must play an important role in preventing hybridization (Marchand 1984).

Diabaté et al. (2009) demonstrated the intricacy of the behavioral components of the speciation process, which may help create novel vector control approaches. However, the question of how this isolation mechanism arises remains unanswered. (Diabaté et al. 2009). Differences in geographical or temporal characteristics related to swarming might help avoid interactions between males and females of different species in a sympatric environment. Further studies should compare premating reproductive isolation between sibling species and forms.

### Pheromones and Swarming

Chemical interactions between animals and their environments are mediated by substances released by one individual and received by another. This type of communication is closely linked to many animal behaviors including mating and aggregation (Brezolin et al. 2018). The chemical ecology of mosquitoes involves insect–plant (repellents and attractants involved in the feeding of larvae and adults), insect–host (attraction to human or mammals), insect–insect (chemical contact among adults), and insect–environment (attraction to oviposition sites) interactions (Lees et al. 2014).

Pheromones are important for mating behavior in many dipteran species, acting as a long-distance attractant to bring males and females together, as well as a means of species identification. The sex pheromones produced by vector organisms directly impact the success of SIT, as attractant compounds are useful for trapping and for modifying swarming behavior (Lees et al. 2014).

Aggregation pheromones promote the formation of animal groups (e.g., males and females of a given species). It provides benefits to individuals such as group living. However, aggregation pheromones may also be used as a response to eavesdropping conspecifics (Wyatt 2003). A five-component blend has been shown to act as an aggregation pheromone in *An. gambiae* and *An. arabiensis* and it increases mating in *An. funestus*, *An. coluzzii*, and *An. merus* (Mozūraitis et al. 2020). In one study, these species produced five identical volatile compounds in the laboratory, namely, octanal, 3-hydroxi-2-butanone (acetoin), 6-methyl-5-hepten-2-one (sulcatone), decanal, and nonanal—at significantly higher quantities during swarming than during nonswarming (Mozūraitis et al. 2020).

To date, no male sex pheromones have been identified in *An. stephensi*, *An. coluzzii*, or *An. gambiae* s.s. (Gendrin 2017, Poda et al. 2020), and there are no known volatile sex pheromones in the *An. gambiae* complex (which includes at least seven genetically distinct species) (Poda et al. 2020). *An. gambiae* males in natural swarms do not respond to females crushed on filter paper or to live females in a net cage (Charlwood et al. 2002a). Females are naturally drawn to male aggregation sites, presumably in response to long-range pheromones emitted by males, although this has yet to be demonstrated (Poda et al. 2020). Cross-population mating studies have indicated that a preference for assortative mating is a female phenomenon (Aboagye-Antwi et al. 2015) and is unlikely to be chemically based (Poda et al. 2020).

The combined impact of visual and chemical cues on swarm formation warrants further investigation (Wooding et al. 2020), as this could reveal the factors that control aggregation behavior and help identify aggregation pheromones in other mosquito species (Vaníčková et al. 2017). An aggregation pheromone could also be used to lure mating males and females into baited traps. Unlike lethal pesticides, all of these methods may be less susceptible to acquired resistance. This underlying biology of male anopheline mosquitoes thus provides numerous untapped and underutilized potential methods for improved studies and practical approaches to limit the substantial harm caused by these hazardous insects (Mozūraitis et al. 2020).

### Circadian Clock and Swarming

Light intensity, day–night cycle, and temperature govern the circadian rhythmicity of physiology, biochemistry, and behavior in most organisms (Sakai and Ishida 2001, Hurley et al. 2016). Mosquito physiology and activity are rhythmically regulated according to the time of day (Sawadogo et al. 2014).

The circadian clock of eukaryotes is cell-autonomous and comprises transcriptional–translational feedback loops that take place over 24 hr (Rund et al. 2013). The locomotor activity and eclosion of *Drosophila melanogaster* are regulated by a central oscillator involving clock genes including *period* (*per*) and *timeless* (*tim*) (Rosato et al. 1997, Scully and Kay 2000). A global transcription analysis revealed that clock genes are linked to swarming activity in the male mosquito. In the laboratory, *Anopheles* males show peak flight activity in the evening, and knockdown of *per* or *tim* gene expression in *An. stephensi* males substantially reduces flying (Wang et al. 2021). In *An. stephensi* male mosquitoes injected with *Drosophila* homologs of *per* or *tim*, maximum swarm height and size (i.e., the number of swarming males) decrease. These findings provide molecular-level evidence for the circadian regulation of swarming and mating behavior in male *Anopheles* (Wang et al. 2021). Moreover, many genes in *An. gambiae* show rhythmic expression only in response to an environmental light/dark cycle, which implies that gene expression is directly regulated by light (Rund et al. 2013).

Temperature also influences the circadian clock (Lamba et al. 2014) and mosquito behavior; mating in *Anopheles* is significantly inhibited as low (19°C) and high (34°C) temperatures relative to the optimum temperature of 27°C (Wang et al. 2021). The regulation of clock genes during swarming and its impact on mating behavior remain to be determined.

### Sugar Feeding and Swarming

Sugars and water in plant fluids are a common source of energy for mosquitoes (Clements 1999). Most mosquitoes’ carbohydrate and lipid reserves at emergence are only adequate to sustain life for a few days, and both males and females feed on plant sugars to obtain energy for swarming and mating (Clements 1999). The only food source for male mosquitoes is plant nectar, so their survival, insemination rates, and swarming ability all depend on nectar availability (Gary et al. 2009, Ebrahimi et al. 2018).

The preference of *An. gambiae* for specific sugar sources is governed by chemical signals, which explains the congregation of males at a variety of flowering plants (Gouagna et al. 2010). Nectar-producing plants near the site of breeding and other activities of adult *An. gambiae* s.s. supply males with nutrients and energy for swarming, which increases the probability of females being inseminated (Gary and Foster 2001). Several plant species in sub-Saharan Africa serve as nectar sources for *Anopheles* (Gouagna et al. 2010, Müller et al. 2010). *An. gambiae* s.s. males predominantly congregate at five plants: flowering *Mangifera indica* L. (Anacardiaceae), *Delonix regia* (Fabaceae), *Thevetia neriifolia* Juss (Apocynaceae), *Senna siamea* (Fabaceae), and *Cassia sieberiana* (both Fabaceae) (Gouagna et al. 2010). *An. arabiensis* males are able to distinguish between possible sugar sources in their native habitat: *Stachytarpheta urticifolia* (Verbenaceae) and *Duranta erecta* (Verbenaceae) are the preferred sugar sources whereas *S. siamea*, *Amaranthus viridis* (Amaranthaceae), and *Centratherum punctatum* (Asteraceae) are the least preferred (Gouagna et al. 2014). Meanwhile, both sexes of *An. gambiae* s.s. favor *Senna didymobotrya*, *S. occidentalis* (both Fabaceae), *Lantana camara* L. (Verbenaceae), and *Parthenium hysterophorus* (Asteraceae), which are thought to produce attractive volatiles (Manda et al. 2007, Nikbakhtzadeh et al. 2014). However, when male mosquitoes are given extrafloral nectar from *M. esculenta*, their mean survival does not vary significantly from those fed a 50% sucrose diet, which is close to the sucrose concentration in nectar (Gary and Foster 2004).

Mosquitoes can distinguish between rich and poor sugar sources, allowing them to select plants with higher glycogen, lipid, and protein contents (Yu et al. 2018). More attractive plants not only elicit higher rates of sugar consumption but also seem to provide more energy (Gouagna et al. 2014). These findings highlight the importance of selective plant feeding for efficient energy acquisition, which is critical for the survival of *An. arabiensis* in sometimes nutritionally sparse and intermittent habitats and is a presumed indicator of fitness during young adulthood. Differences in energy levels among individuals may be attributable to variation in sugar intake rates and quantity, which can affect energy metabolism; or to the distinct sugar profiles of nectars that differentially stimulate male chemoreceptivity (Gouagna et al. 2014). Detailed information on the relationship between nectar production in plants and sugar feeding behavior in mosquitoes and the energetic benefits thereof, is currently lacking.

Feeding on various natural sugar sources affects physiological development and thus, the life history of mosquitoes (Reisen et al. 1983, 1986). *An. coluzzii* males fed papaya juice live longer than those fed mango and banana juices, and mosquitoes fed mango juice are less likely to participate in swarming, possibly due to an inadequate amount of energy and decreased competitiveness (Nignan et al. 2020).

A male mosquito can mate several times during its lifetime (Ekechukwu et al. 2015), and energy consumption during swarming flight is exceptionally high (Gary et al. 2009, Maïga et al. 2014). Swarming behavior in male *Anopheles* uses approximately 50% of the reserves of glycogen and sugar, which is the primary source of energy used in flight (Maïga et al. 2012, Yahouédo et al. 2014). *An. gambiae* s.s. has 6% higher sugar and glycogen contents than *An. coluzzii*; this difference is linked to the effects of the environment on male body size and energy reserves (Maïga et al. 2014). Nocturnal feeding allows males to replenish their energy reserves after each swarming event (Gary et al. 2009, Maïga et al. 2014, Nignan et al. 2020). At present, there is limited evidence linking the feeding of male *Anopheles* on sugars from specific plant species to their swarming behavior.

### Swarming and the Abiotic Environment

Identifying the environmental variables that govern mating activity can inform the development of effective strategies for controlling mosquito populations (Bimbilé Somda et al. 2018). Swarming and mating in *An. arabiensis* are influenced by climatic variables that vary across season including temperature, sunset time, day length, and amount of rainfall (Bimbilé Somda et al. 2018).

The duration of swarming and number of mosquitoes per swarm are inversely related to total rainfall and rain frequency; the shortest swarm times and smallest swarms are observed at the peak of the rainy season, while larger and longer-lasting swarms occur during the dry season. The causes of low reproductive activity during the rainy season are not fully understood (Bimbilé Somda et al. 2018).

Dusk falls at different times throughout the year, which has an effect on swarming in *Anopheles*. In Burkina Faso, swarms often appear at or following sunset from late July to early October and sometimes before sunset during the rest of the year. There is a strong association between swarming start and end times and the time of sunset. However, the change in temperature accompanying sunset is only weakly associated with the beginning and end of swarming activity (Bimbilé Somda et al. 2018).

The host-seeking flight activity of female *An. gambia* s.s. is reduced by the decrease in ambient light level at dusk and increased during the middle and end of the night (Sheppard et al. 2017). In females of Sahelian *An. coluzzii*, insemination rates range from 64% from January–February to 94% in June. An increase in rate during the dry season is to be expected, as it corresponds to the seasonal variation in swarming activity of *An. coluzzii* males. Some females enter estivation before being inseminated (Yaro et al. 2012). Overall, many environmental factors, such as temperature, humidity, precipitation, moon cycle, and gravity directly affect mosquito swarming behavior. However, it is not known whether a single or combination of multiple factors influences behavioror whether the response of mosquitoes to these factors differs according to the environment.

## Conclusion

This review summarizes the current state of knowledge regarding mosquito swarming behavior. Targeting swarming sites to reduce mosquito populations is an effective way to control the spread of mosquito-borne diseases such as malaria. Methods for controlling mosquito populations without insecticides are desired to protect local ecosystems and the environment. There are many avenues for future research on swarming and mating in *Anopheles*. The use of devices such as video cameras and sizeable experimental field cages can yield more quantitative and empirical data. By identifying the wing beat signals and visual markers of a swarm, specific mosquito behaviors can be targeted to reduce population sizes. Data on swarm height and the start and end times of swarms are needed for more *Anopheles* species; and swarm detection mechanisms in both sexes have yet to be characterized. In addition, the mechanisms used by females to avoid mating with males from other species or molecular forms warrant further investigation.
